# Impulsivity in cerebellar ataxia: an online, multidimensional assessment

**DOI:** 10.1007/s00702-025-03020-z

**Published:** 2025-09-13

**Authors:** Brooke Chasalow, Yakov Flaumenhaft, Yael De Picciotto, Chi-Ying R. Lin, Leila Montaser-Kouhsari, William Saban

**Affiliations:** 1 Center for Accessible Neuropsychology, Sagol School of Neuroscience, Tel Aviv University, Tel Aviv, 69978 Israel; 2 Department of Occupational Therapy, Gray Faculty of Medical & Health Sciences, Tel Aviv University, Tel Aviv, 69978 Israel; 3 The School of Psychological Sciences, Gershon H. Gordon Faculty of Social Sciences, Tel Aviv University, Tel Aviv, 69978 Israel; 4 Department of Neurology, Baylor College of Medicine, Houston, TX USA; 5 Department of Neurology, Brigham and Women Hospital, Harvard University, Boston, MA USA

**Keywords:** Cerebellar ataxia, Cerebellum, Impulsivity, BIS-11, Delay discounting, Reward

## Abstract

While considered a motor control structure, the cerebellum contributes to non-motor functions, including impulsivity. However, whether it contributes to impulsivity in a domain-specific manner remains unknown. Studies on cerebellar ataxia (CA), a common model for cerebellar dysfunction, typically have small sample sizes, limiting robustness. In a multicenter cross-sectional study, we investigated the cerebellum’s role in various forms of impulsivity by comparing large cohorts of CA to age- and education-matched neurotypical healthy (NH) controls. Additionally, to examine the ability to identify individuals with CA using impulsivity features alone, we developed supervised machine learning (ML) models. In experiment 1 (CA = 140, NH = 136), impulsivity was assessed using the BIS-11 questionnaire. In experiment 2 (CA = 110, NH = 107), performance-based impulsivity was assessed using the MCQ-27, evaluating delay discounting in monetary decision-making. Two ML models—Logistic Regression and Random Forest—were utilized to classify disorder status (CA/NH). The CA group showed higher BIS-11 scores (*p* = 0.001), indicating higher impulsivity, driven by motor (*p* < 0.001) and attention (*p* = 0.002) impulsivity. However, the CA group exhibited lower non-planning impulsivity (*p* = 0.014). In the MCQ-27, the CA group showed lower k-values (*p* < 0.005), indicating reduced impulsivity in monetary decisions. Both ML models demonstrated strong classification performance (AUC ≥ 0.85) in independent datasets. This study highlights the cerebellum’s selective role in impulsivity. We found higher motor and attentional impulsivity in CA alongside lower non-planning and decision-making impulsivity. This suggests a unique impulsivity profile in CA that may indicate a compensatory mechanism for future events. ML models demonstrated high classification performance, suggesting impulsivity is a core non-motor feature of CA.

## Introduction

While the cerebellum, a phylogenetically ancient brain region, has been traditionally viewed as a motor structure, converging evidence suggests that its function extends beyond motor control (Middleton and Strick 2000; Schmahmann 2004; Saban et al. 2025; Flaumenhaft et al. 2025; Ravizza et al. 2006; Strick et al. 2009; Guell et al. 2018; Hull 2020; Saban et al. 2024). Support for this broader cerebellar role can be found in its bidirectional connectivity with the neocortex (Watson et al. 2014; Caligiore et al. 2016; Bostan and Strick 2018), neuroimaging studies showing cerebellar activation in cognitive tasks (Stoodley et al. 2012; Walz et al. 2015), and cognitive deficits in individuals with cerebellar pathology (McDougle et al. 2022; Saban et al. 2024). A growing body of research revealed cerebellar involvement in various cognitive abilities, including executive function, and even mathematical processes (Schmahmann 2004; Saban et al. 2025; Ravizza et al. 2006; Strick et al. 2009; Barth et al. 2010; Flaumenhaft et al. 2025; Caligiore et al. 2016; Argyropoulos 2016; Bostan and Strick 2018; Saban and Gabay 2023; Saban et al. 2024; Daniel et al. 2025). While these studies support a cerebellar role in non-motor functions, further direct evidence, and a robust theoretical framework are needed to clarify its precise contributions to higher cognitive and behavioral function. One such area that warrants further investigation is the impact of cerebellar dysfunction on particular aspects of impulsivity.

Impulsivity can be defined as a “predisposition toward rapid, unplanned reactions to internal or external stimuli without regard to the negative consequences of these reactions” (Moeller et al. 2001). Despite the initial tendency to associate impulsivity with top-down cognitive control – focusing on cortical structures – impulsivity recruits the interaction of a neural network, involving both cortical and sub-cortical regions (Whelan et al. 2012; Frost and McNaughton 2017; Mehta et al. 2023). Previous studies have focused on the fronto-basal ganglia network and the dopaminergic system in impulsivity (Weintraub and Claassen 2017; Lansdall et al. 2017; Johnson et al. 2017; Frost and McNaughton 2017; Mehta et al. 2023).

However, increasing evidence suggests the potential role of the cerebellum in impulsivity (Miquel et al. 2019). Concurrently, the frontal lobe and basal ganglia, known for their role in inhibition and reward-related processing, has strong connectivity with the cerebellum (Middleton and Strick 2000; Bostan and Strick 2018; Flace et al. 2021; Yoshida et al. 2022). Neurophysiological findings from animal models demonstrated the cerebellum’s involvement in modulating reward processing (Heffley et al. 2018; Kostadinov et al. 2019; Carta et al. 2019; Sendhilnathan et al. 2020). Within the cerebellum, granule neuronal firing patterns encode reward processes and anticipation (Wagner et al. 2017), while climbing fiber firing patterns convey information related to reward prediction error (Kostadinov et al. 2019). Moreover, Purkinje cell firing patterns have been linked to reward processing via reinforcement learning (Ohmae and Medina 2015). Furthermore, neuroimaging evidence has also indicated cerebellar contributions to impulsivity. For instance, stronger cerebellar–basal ganglia connectivity has been associated with higher impulsivity in Parkinson’s disease (PD), as reflected by elevated Barratt Impulsiveness Scale (BIS) scores. In addition, PD patients with impulse control disorders (ICDs) exhibited increased striatal–cerebellar connectivity but reduced parietal–cerebellar connectivity, as well as distinct patterns of cerebellar activation during probabilistic decision-making (Ruitenberg et al. 2018, 2022). These findings, together with more recent work (Warden et al. 2025), point towards a multidimensional view of impulsivity, with distinct impairment patterns and underlying neural mechanisms that differentiate motor/action from choice-related impulsivity.

This corresponds with broader research demonstrating that impulsivity is not a unitary construct but a multifaceted phenomenon comprising multiple components (Stanford et al. 2009; da Matta et al. 2012; Frost and McNaughton 2017; Jauregi et al. 2018a; Lai et al. 2023). Accordingly, its assessment requires multiple complementary measures rather than reliance on a single tool. To address this, we employed two different measures of impulsivity in the present study. The Barratt Impulsiveness Scale (BIS-11)(Patton et al. 1995) is a widely used self-report test for assessing trait impulsiveness, measuring three subdomains of impulsivity: Motor, attentional, and non-planning (Stanford et al. 2009). In addition, in the context of decision-making, another form of impulsivity is delay-discounting (DD). DD is the tendency to devalue rewards as the delay in receiving them increases, reflecting a preference for smaller, immediate rewards over larger, delayed ones (Godefroy et al. 2023; Kirby et al. 1999). Individuals who tend to prefer the smaller, more immediate rewards are considered to have higher DD (higher k value), reflecting greater decision-making impulsivity (Kirby et al. 1999; da Matta et al. 2012; Pennisi et al. 2023). One common paradigm to assess DD is the Monetary Choice Questionnaire (MCQ) (Kirby and Maraković 1996), which has been extensively validated (Kirby et al. 1999; Marco-Pallarés et al. 2010; Kaplan et al. 2016; Gray et al. 2016; Thrailkill et al. 2022; da Matta et al. 2012; Rung and Madden 2018; Pennisi et al. 2023). Based on the participant’s performance in the MCQ, a DD rate is calculated (k value). Lower k values indicate a preference for higher, later rewards rather than smaller, immediate ones (Kirby et al. 1999; da Matta et al. 2012; Kaplan et al. 2016).

One common method to investigate cerebellar contributions to cognitive-behavioral processes is studying Cerebellar Ataxia (CA) patients (Saban and Ivry 2021; Lai et al. 2023; Saban et al. 2024). CA is a rare (< 0.01%) neurodegenerative disorder of the cerebellum primarily manifested by impairment of motor control. Studies on CA demonstrated cognitive alterations in these patients (Schmahmann 2004; Smeets and Verbeek 2014), but frequently these studies have small cohorts with less than 20 participants (Olivito et al. 2018; Wang et al. 2018; Tang et al. 2020). Indeed, in recent years, more studies indicated the potential involvement of the cerebellum in impulsivity and reward mechanisms by studying CA patients, further implying that in humans, an intact cerebellum is important for reward processing (Amokrane et al. 2020a, b; Chen et al. 2022; Lai et al. 2023, 2024; Lin et al. 2024a). For instance, recent work(Chen et al. 2022) assessed impulsivity in CA, Parkinson’s disease (PD), and neurotypical healthy (NH) groups using the BIS-11 and found the total score of the CA group (and PD) to be higher than the NH group (Patton et al. 1995). Additionally, a case study of five CA patients reported that all five individuals demonstrated various impulsive behaviors (Amokrane et al. 2020a). Another study compared impulsive behaviors in CA to NH controls using a self-report questionnaire and found more impulsivity in the CA group in specific domains, such as gambling and excessive medication use, but not in eating, buying, and sex (Amokrane et al. 2020b). While multiple studies have demonstrated altered DD in neurodegenerative conditions (Pennisi et al. 2023; Godefroy et al. 2023), to our knowledge, no previous study has examined DD in CA. Thus, employing this measure in the current study provides unique and additional insight into the impulsivity of CA patients.

The above-mentioned scarce body of literature indicates the potential involvement of the cerebellum in impulsivity. However, the precise alterations in different aspects of impulsivity following human cerebellar pathology remain poorly understood. To address this gap, we examined various aspects of impulsivity in large-scale CA and NH cohorts, employing both the BIS-11 and a DD task. To overcome challenges faced by the small samples of previous studies, we employed an online neuropsychological testing method. This approach enabled us to recruit large cohorts of age- and education-matched CA and NH participants. In doing so, we address previous concerns about limited sample sizes and restricted generalizability in this rare clinical population, promoting potential robustness of the findings. Based on recent work (Chen et al. 2022), we hypothesized that the CA group would have higher levels of impulsivity than the NH group.

## Methods

### Participants

**Fig. 1:**
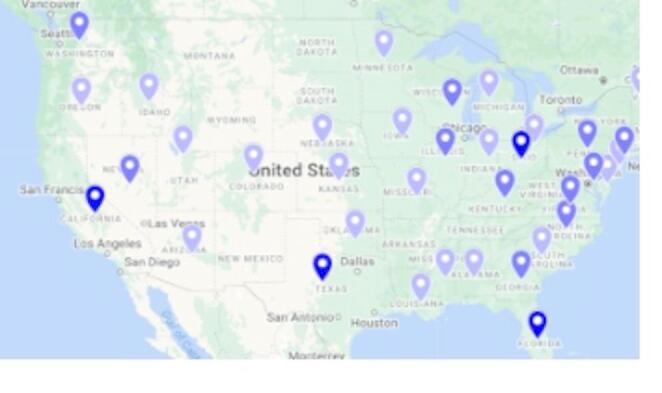
Geographical marker map of cerebellar ataxia participants from 39 states

In this cross-sectional study, we collected data from large cohorts through collaboration across multiple centers. Figure 1 presents a geographical marker map of cerebellar ataxia participants from 39 states across the USA. The required sample size was determined based on prior work directly comparing CA and NH participants on the BIS-11 total score (Chen et al. 2022). A power analysis (α = 0.05, power = 0.95, Cohen’s d = 0.698) indicated that a minimum of 45 participants per group would be sufficient to detect group differences. Given that each of our study groups included more than 100 participants, the sample sizes provided adequate statistical power for the planned analyses.

In Experiment 1, 140 individuals diagnosed with CA and 136 age, education, and sex-matched NH participants completed the BIS-11. In Experiment 2, 112 individuals with CA and 109 matched NH participants completed the DD task. In the DD task, four participants were excluded (2/group) based on a consistency score of $\:\le\:\:$70% (Xu et al. 2022). Thus, 110 CA and 107 NH participants were included in the final analyses. A total of 89 participants with CA and 107 NH completed both the BIS-11 and the DD task.

Table 1 presents the demographic and clinical characteristics of all study cohorts. Participants with a confirmed diagnosis of CA were recruited through the Center for Accessible Neuropsychology (CAN) clinical database, which includes individuals previously assessed at CAN or those who responded to targeted online advertisements disseminated through CA-specific organizations (e.g., the National Ataxia Foundation). Exclusion criteria included a history of other neurological conditions (excluding CA), psychiatric disorders, learning disabilities, or significant visual or auditory impairments. Participants diagnosed with multiple system atrophy, brain tumors, or a history of ischemic or hemorrhagic stroke were also excluded. In addition, given the established association between dopamine and impulsivity, individuals using dopaminergic medications (e.g., carbidopa/levodopa, dopamine agonists) were not eligible for participation. We did not systematically control for all non-dopaminergic medications. NH participants were recruited via the Prolific online platform (Palan and Schitter 2018), using demographic matching procedures to align with the CA group in terms of age, years of education, and gender distribution, while also ensuring the absence of self-reported cognitive impairment. All participants were aged 18 or older and were required to understand and sign informed consent.

### Neurological and neuropsychological assessment

Similar to previous studies (Algon et al. 2025; Saban and Ivry 2021; Binoy et al. 2023; Picciotto et al. 2024; Gilad et al. 2025; Schönfeldová et al. 2025), we followed the online neuropsychological testing protocol, called iPONT: International Protocol for Online Neuropsychological Testing for CA subjects. After signing the informed consent, the experimenter obtained the CA participant’s medical history, collecting information about age at diagnosis, medication, primary symptoms, genetic subtype, diet, and other neurological or psychiatric conditions. The experimenter administered the Montreal Cognitive Assessment test (MoCA)(Nasreddine et al. 2005; Binoy et al. 2023; Picciotto et al. 2024; Gilad et al. 2025; Schönfeldová et al. 2025) as a brief evaluation of cognitive status. The MoCA was administered only to the CA group as a cognitive screening tool to exclude individuals with potential cognitive impairments that could confound impulsivity-related performance. NH participants were pre-screened via the Prolific platform (Palan and Schitter 2018) to ensure no self-reported cognitive or neurological conditions, and therefore, MoCA was not administered to this group. The CA cohort displayed a broad range of disease severity and disorder durations (see Table 1). Finally, the experimenter administered the Scale for Assessment and Rating of Ataxia (SARA) (Schmitz-Hübsch et al. 2006; Picciotto et al. 2024; Algon et al. 2025) as an evaluation of disorder severity. The participants had diverse^1^ known, genetically or etiologically confirmed types of CA, such as spinocerebellar ataxia (SCA) 1, 2, and 6, and 73 had CA of unknown etiology. The CA and NH groups did not significantly differ in age or years of education (*p* > 0.05). These variables were initially included as covariates; however, they did not exert a significant effect on the results. Therefore, the data was collapsed across these variables.

**Table 1:** Demographic and medical summary of both groups in experiments 1 and 2. Mean [SD]

Measure	Group	*n*	Age	Females %	Years of Education	MoCA	Disorder Duration	SARA
BIS-11	Cerebellar Ataxia	140	55 [13.5]	62	16.3 [2.6]	26.4 [2.6]	7.7 [7.6]	13.9 [5.9]
	Neurotypical Healthy	136	56.9 [12.2]	61	15.8 [2.6]	na	na	na
Delay discounting	Cerebellar Ataxia	110	55.9 [13.9]	64	16.4 [2.6]	25.7 [3.1]	8.6 [8.2]	14.6 [6.4]
	Neurotypical Healthy	107	55.7 [12.5]	59	15.7 [2.7]	na	na	na
Both	Cerebellar Ataxia	89	56 [13.8]	62	16.6 [2.6]	25.8 [3]	8.5 [8]	14.5 [6.1]
	Neurotypical Healthy	107	55.7 [12.5]	58.8	15.7 [2.7]	na	na	na

### Procedure

The experiment was programmed in Gorilla Experiment Builder (Anwyl-Irvine et al. 2020) and designed to be compatible with personal computers. Participants completed the Barratt Impulsiveness Scale version 11 (BIS-11) questionnaire and the delay discounting (DD) task (MCQ-27) presented in a mixed random order. Text-based prompts (text) were presented at the center of the screen as black characters on a white screen during both the BIS-11 (as Likert-scale statements; i.e. “I plan tasks carefully”) and the MCQ-27 (as binary monetary choice items; i.e. “Which would you prefer to receive?”). Participants were invited by email to participate in an experiment. The email provided an overview of the experimental task and included a link that could be clicked to initiate the experimental session. The link was associated with a unique participant ID, providing a means to ensure that the data were stored in an anonymized and confidential manner.

### Main measures

#### BIS-11

We utilized the BIS-11 (Patton et al. 1995). This 30-item questionnaire is scored on a 4-point Likert scale from 1 (rarely/never) to 4 (almost always). Factor analysis indicates three impulsiveness traits (subscale): Motor impulsivity (acting without thinking), attentional impulsivity (inability to concentrate or focus), and non-planning impulsivity (lack of planning) (Patton et al. 1995). The BIS-11 total score ranges from 30 to 120 with higher scores representing higher impulsiveness. A total score of 72 or higher classifies individuals as highly impulsive (Patton et al. 1995) The BIS-11 has been extensively validated and shown to be a reliable and valid measure of impulsivity (Stanford et al. 2009).

#### Delay discounting

To assess DD, we used an online version of the MCQ with 27 dichotomous choice items between a smaller immediate reward versus a larger delayed reward. The task is preconfigured to estimate an individual’s DD rate across three delayed reward sizes: Small ($25–35), medium ($50–60), and large ($75–85) along with an overall DD rate (Gray et al. 2016). Participants were instructed to approach each decision as if it were real. Each item of the MCQ was presented in mixed random order. Past research supports the validity of hypothetical DD measures, as they yield similar discounting rates to real-reward scenarios (Johnson and Bickel 2002; Madden et al. 2003; Lagorio and Madden 2005; Odum 2011; Kaplan et al. 2016). Nevertheless, to maximize reliability and incentivize real-world choices, participants were informed they would receive 10% of one of the options they selected (Godefroy et al. 2023). In line with previous studies, rates of DD were calculated using the hyperbolic formula:

$\:V=\:\frac{A}{1+kD}$, where V is the present value of the delayed reward A after delay D, and k represents the delay discount rate (Kaplan et al. 2016; Godefroy et al. 2023). Higher k values indicate a steeper DD rate and greater impulsivity in monetary decision-making. Since the distribution of raw k values is skewed, logarithmic transformations (log k values) were used to approximate a normal Gaussian distribution for statistical analyses (Landes et al. 2010; Mitchell et al. 2015; Kaplan et al. 2016).

### Statistical analysis

All analyses were conducted using the JASP or R software (JASP Team 2024). We conducted independent sample t-tests to compare scores between the two groups (BIS-11 total and second-factor scores, log k, and magnitudes log k). We used Holm–Bonferroni corrections for multiple comparisons. We conducted exploratory correlation analyses between variables of interest, including the relationships between impulsive measures and the MoCA, disorder severity, and disorder duration. As these were exploratory analyses, we did not correct for multiple comparisons.

### Machine learning models for the classification of CA and NH

We utilized Machine learning (ML) classifiers aimed to evaluate the robustness of our results by examining the predictive power of impulsivity features alone in differentiating between CA and NH participants. All models were developed and implemented using the scikit-learn package in Python (Pedregosa et al. 2011). We used two ML models: (1) Logistic Regression (LR) with LASSO regularization and (2) Random Forest (RF) classifier. These ML models were used to binary classify CA or NH based on the impulsivity features: second-order factors of BIS-11 (attention, motor, and non-planning) and DD measures by reward size (small, medium, and large k values, consistency score, and percent of “now”). To minimize multicollinearity, the total scores (BIS-11 and log K) were not included, and LASSO regularization was applied to penalize redundant predictors.

To assess the ML model’s generalizability, we applied 10-fold cross-validation, a procedure in which the dataset is randomly divided into ten equal-sized folds. In each iteration, the ML model is trained on nine folds and tested on the remaining one, ensuring that every observation serves as a test case. This technique allowed us to assess whether the ML models can effectively predict the classification of participants based on impulsivity features in a new, independent dataset. Accordingly, the data was split into train and test datasets (LR: 90:10; RF: 80:20). All features were normalized using Z scores to avoid bias.

The ML models’ classification performance was evaluated using two performance metrics: Area Under the Receiver Operating Characteristic Curve (AUC-ROC) and accuracy (including precision and recall). The ROC curve plots the True Positive Rate (TPR) against the False Positive Rate (FPR) at various threshold settings. AUC values range from 0 to 1, with 0.5 representing random guessing and 1 indicating perfect classification.

## Results

### Experiment 1—BIS-11

**Fig. 2:**
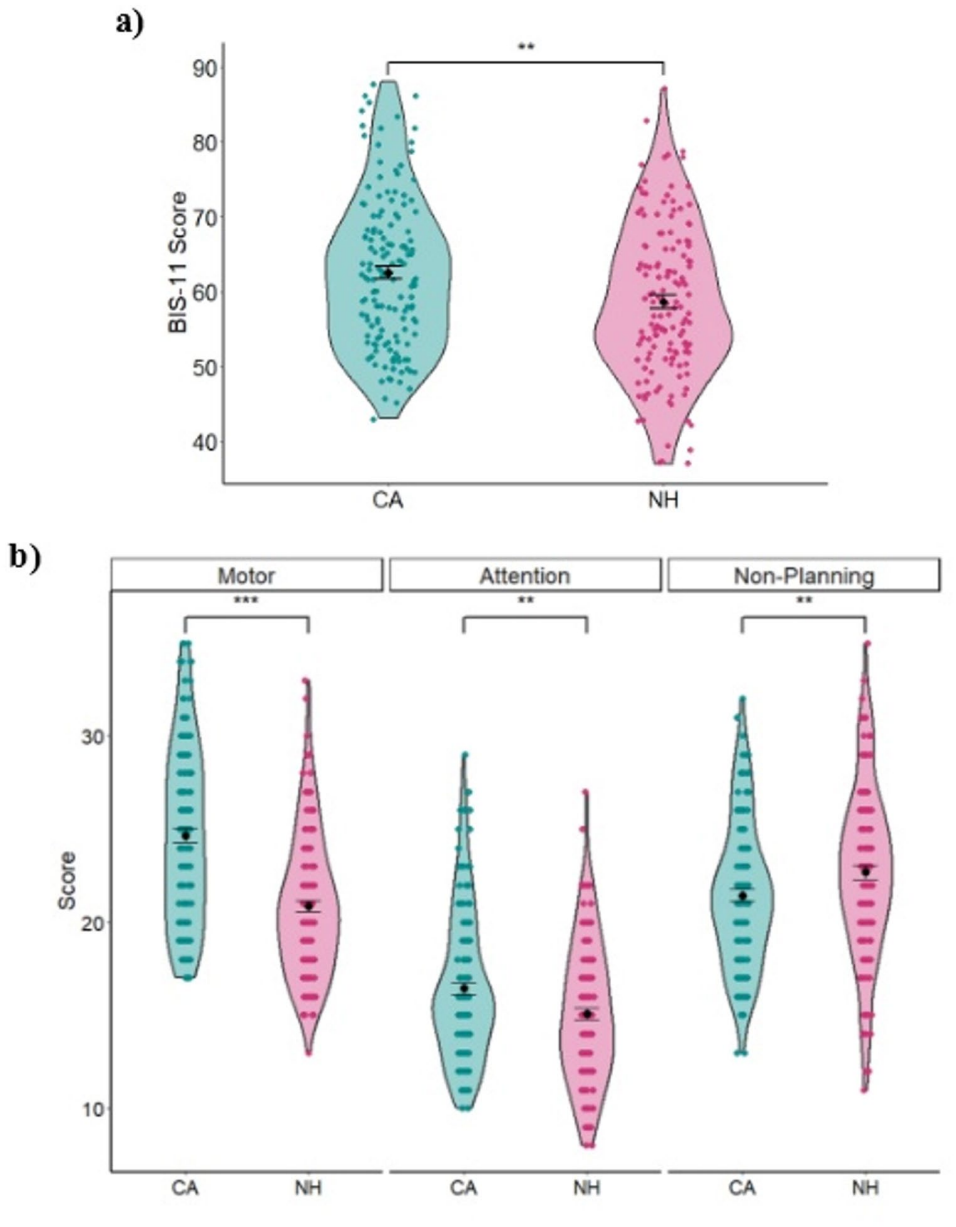
**a** Barratt Impulsiveness Scale (BIS-11) scores for the cerebellar ataxia (CA) and neurotypical healthy (NH) groups. **b** Subdomains by group **p < 0.05; ***p < 0.001

As predicted, the CA group obtained a significantly higher score than the NH group in the BIS-11 total score (CA = 62.4 ± 0.8 vs. NH = 58.3 ± 0.9, t(274) = 3.342, *p* = 0.008, Cohen’s d = 0.402, Fig. 2a). When comparing the groups in the subdomains, we found a significant difference in all three subdomains. In the motor and attention factors, CA scored significantly higher than the NH group (Motor: CA = 24.6 ± 0.4 vs. NH = 20.7 ± 0.3, t(274) = 7.57, *p* < 0.007, Cohen’s d = 0.911; Attention: CA = 16.4 ± 0.3 vs. NH = 15 ± 0.3, t(274) = 3.164, *p* = 0.006, Cohen’s d = 0.381, Fig. 2b). However, in the non-planning factor, CA scored significantly lower than the NH group (CA = 21.3 ± 0.3 vs. NH = 22.6 ± 0.4, t(274) = −2.473, *p* = 0.014, Cohen’s d = −0.298, Fig. 2b).

### Experiment 2—Delay discounting

**Fig. 3:**
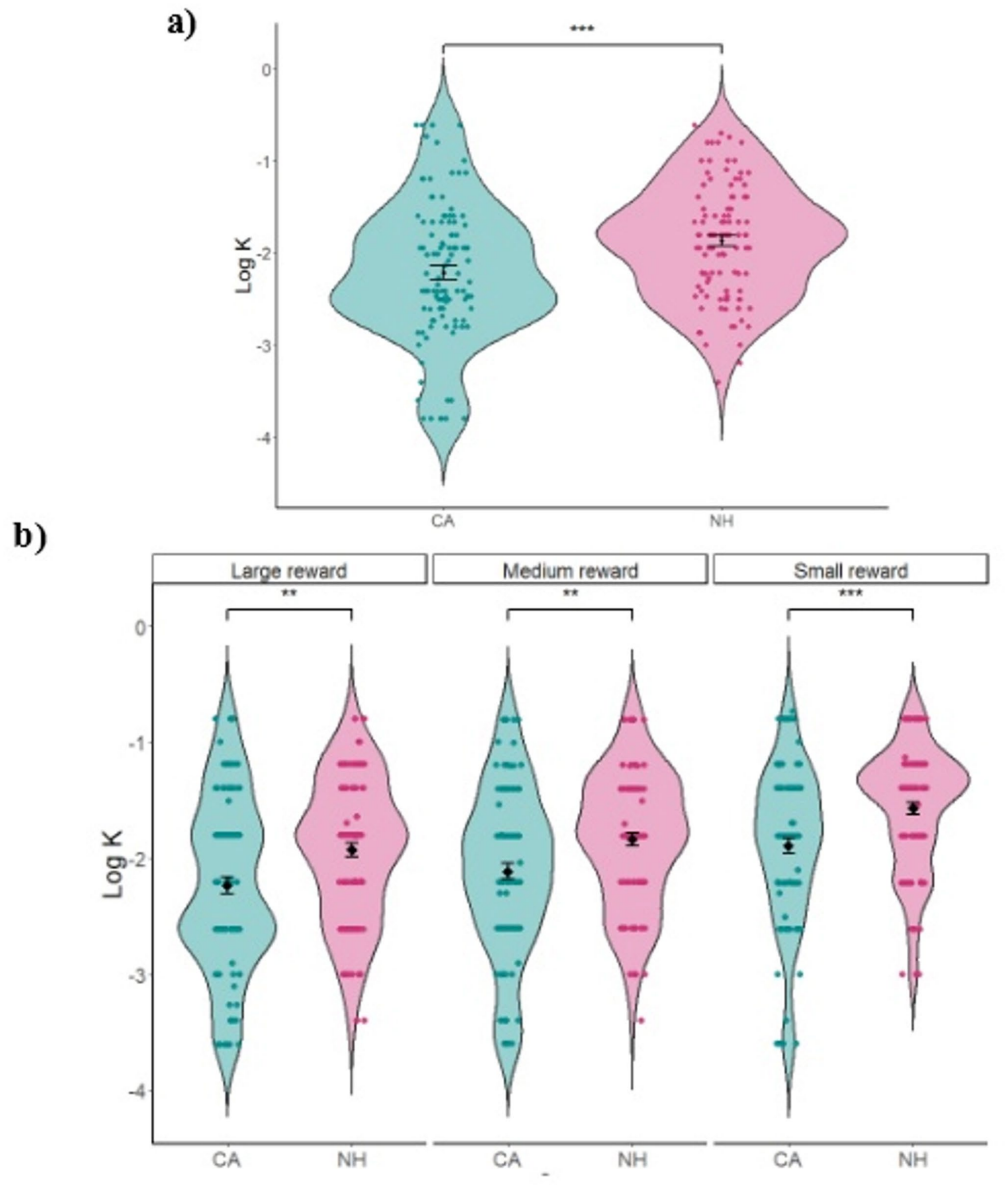
**a** Delay discounting rates values (log k) for the cerebellar ataxia (CA) and neurotypical healthy (NH) groups. **b** Delay discounting rates for the three monetary amount magnitude **p < 0.05; ***p < 0.001

Both non-planning impulsivity and DD reflect reduced consideration of the future. Based on our findings in Experiment 1, we anticipated that in the DD task, the CA group should have lower k values. This hypothesis is supported by literature showing that lower non-planning impulsiveness is correlated with lower delay discounting rates (de Wit et al. 2007; Mobini et al. 2007). Our results demonstrated that the CA group indeed had significantly lower log k values compared to the NH group (*t*(215) = −3.683, *p* < 0.006, Cohen’s d = −0.500, Fig. 3a), suggesting a lower discounting rate. A similar pattern was found across all the monetary amount magnitudes (Small: *t*(215) = −3.964, *p* < 0.005, Cohen’s d = −0.538; Medium: *t*(215) = −3.151, *p* = 0.004, Cohen’s d = −0.428; Large: *t*(215) = −3.353, *p* < 0.004, Cohen’s d = −0.455, Fig. 3b).

### Correlation with other cerebellar symptoms

We did not find a significant correlation between cognitive status (i.e., MoCA score) and the BIS-11 total score (*r* = 0.009, *p* = 0.475) as well as the log k value of the DD task (*r* = 0.117; *p* = 0.212). These two analyses suggest that the current impulsivity measures are distinct cognitive-behavioral features of CA.

In addition, ataxia severity (SARA) did not significantly correlate with either the BIS-11 total score (*r* = − 0.006, *p* = 0.961) or log k values (*r* = − 0.224, *p* = 0.097). Interestingly, while there was no significant correlation between disorder duration and BIS-11 score (*r* = 0.019, *p* = 0.845), there was a significant, inverse correlation between disorder duration and log k value (*r* = − 0.212, *p* = 0.029), implying that reduced decision-making impulsivity is more pronounced with disorder progression.

### Machine learning models for the classification of CA and NH

**Fig. 4:**
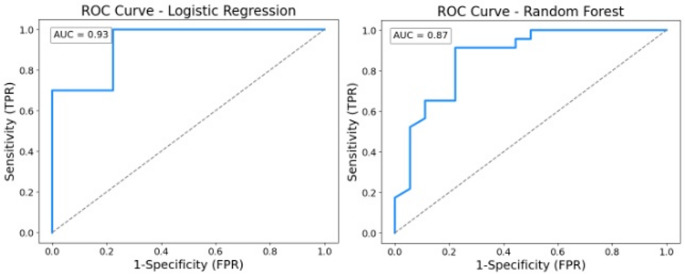
Receiver Operating Characteristic (ROC) Curve for both machine learning models. *TPR* true positive rate, *FPR* false positive rate, *AUC* area under the curve

The Logistic Regression model with LASSO regularization achieved an AUC of 0.93 and an accuracy of 74% (precision = 74%, recall = 74%; see Fig. 4). The Random Forest model yielded an AUC of 0.87 and an accuracy of 83% (precision = 84%, recall = 83%; see Fig. 4). Thus, both ML models demonstrated strong predictive classification performance (CA vs. NH). This suggests ML models can effectively generalize the classification of individuals based on impulsivity features to a new, independent dataset.

## Discussion

Our study explored the role of the human cerebellum in different impulsivity processes, utilizing large cohorts of age- and education-matched CA and NH controls. We employed two complementary measures of impulsivity to examine cerebellar contributions to impulsivity. The use of an online protocol for neuropsychological testing enabled broad recruitment across geographical regions, increasing sample diversity and reducing potential sampling biases that may have confounded earlier studies (Chen et al. 2022).

Consistent with previous findings (Chen et al. 2022), the CA group scored higher overall on the BIS-11 compared to the NH group, indicating higher reports of impulsivity. Of note, the results reveal that higher impulsivity reported by CA individuals may be limited to specific subdomains of impulsive traits. This higher level of impulsivity was found in two of the three domains assessed by the BIS-11: motor and attentional impulsivity. In contrast, for the non-planning domain, the CA group reported lower impulsivity than the NH group. Accordingly, the CA group had a lower DD, indicating more willingness to wait for larger, delayed rewards. These findings converge, as both reports of higher planning and lower DD reflect greater consideration of future outcomes. Impulsivity results from the interaction between decision-making and reward systems, suggesting distinct neural networks might influence its various aspects, with the cerebello-ventral tegmental area excitatory pathway being one example (Lee et al. 2011; Whelan et al. 2012; Carta et al. 2019).

The ML analyses were intended to complement our primary group comparisons by testing whether impulsivity features alone could differentiate between CA and NH participants. The ML analyses aimed to evaluate the robustness of our results by examining the predictive power of impulsivity features in differentiating between CA and NH participants. Using impulsivity features from both experiments, the ML models provided additional support for the above findings, achieving high classification performance (CA vs. NH) based on non-motor features alone. The application of two ML classifiers (Logistic Regression and Random Forest) yielded consistent results, reinforcing the robustness of the findings that impulsivity is associated, and might have predictive power, to CA. The high classification performance demonstrated by the ML models supports the notion that impulsivity is a core non-motor feature of the CA population.

The cerebellum is increasingly recognized for its compensatory effects on non-motor function through connections with the cerebrum and basal ganglia in neurodegenerative disorders (Kawabata et al. 2020; Yin et al. 2021; Lin et al. 2024b). Our results, demonstrating higher reports of planning in CA, may represent a subjective compensatory mechanism for the lack of motor control, the major symptom of CA. Prior research has demonstrated that alternative neural pathways can be recruited in highly impulsive individuals to resist immediate rewards and prioritize long-term goals (Diekhof et al. 2011). Furthermore, evidence suggests that the cerebellum plays a critical role in implicit sequencing, particularly in feedforward mechanisms that predict future outcomes. Individuals with CA can compensate for cognitive sequencing impairment, particularly when explicit strategies are available (Morgan et al. 2021). Given that explicit strategies are probably involved in both the BIS-11 and DD, it is plausible that the CA’s current results may derive from a neural network compensation mechanism, allowing the orchestration of impulsivity.

Interestingly, our findings both partially align with and partially diverge from those of previous studies (Chen et al. 2022; Ruitenberg et al. 2002; Ruitenberg et al. 2018; Warden et al. 2025). Our results align with the view that impulsivity comprises distinct domains of action/motor and choice impulsivity (Ruitenberg et al. 2002; Ruitenberg et al. 2018; Warden et al. 2025). The current study findings of higher motor and attentional impulsivity but lower decision-making impulsivity in CA suggest a selective cerebellar role in these domains. Additionally, in both our study and a previous study (Chen et al. 2022), the BIS-11 total score was higher in the CA group compared to the NH group. However, while this previous study found higher non-planning impulsivity, we found lower non-planning in the CA group. Several methodological factors should be taken into account. First, the sample size variation across studies could have played a role: this previous study’s small NH sample (*n* = 40) reported a relatively low total BIS-11 mean score of 53.6 (Chen et al. 2022). In contrast, the norms of the BIS-11 total score (Stanford et al. 2009)–calculated based on a large NH sample of 1577 participants–had a significantly higher (16%) mean score of 62.3. For comparison, our current sample of NH scored 58.3. Along this line, the percentage of NH participants that met clinical thresholds for impulsivity (> 71) was just 1% for this previous study (Chen et al. 2022) whereas this was 16% for the normative sample (Stanford et al. 2009). For comparison, our current samples have bigger than 1% (NH = 11.7%, CA = 18.5%), similar to the norms. Additionally, while CA is primarily characterized as a motor control disorder (Manto et al. 2024) this previous study did not find higher reports of CA in the motor domain of impulsivity. These several discrepancies indicate that the previous study sample may not accurately represent the broader population, potentially underestimating impulsivity levels in the NH population. Indeed, the NH sample in the previous study was recruited exclusively from a single geographical location in the USA, which may have influenced sample diversity and representation. This potential bias underscores the necessity for further research directly comparing NH and CA populations, utilizing larger and more diverse samples, ensuring the results are reflective of the population’s impulsivity levels.

A few limitations should be considered. Since CA patients may also present extracerebellar pathology, examining a subgroup of individuals with spinocerebellar ataxia type 6 (SCA6), a relatively pure form of cerebellar degeneration, would provide more refined insights into the cerebellum’s role in impulsivity. However, although the overall sample size in our study was large relative to previous research on CA and included patients with SCA6 (*n* = 13), this subgroup did not meet the minimum statistical power requirement (*n* = 45) necessary for such analyses. Additionally, although both tools used in this study pointed to similar overall conclusions, the exact relationship between the different dimensions of trait- and performance-based impulsivity still requires clarification (Jauregi et al. 2018b). Moreover, the absence of MoCA data for the NH group precludes direct group comparisons in cognitive status. In the present study, NH participants were pre-screened to exclude cognitive or neurological conditions, and CA participants’ MoCA scores did not correlate with impulsivity measures; these null correlations support the interpretation that the observed group differences reflect specific alterations in impulsivity rather than being driven by general cognitive impairment. Nonetheless, future studies should consider applying consistent cognitive screening tools across all groups. Another consideration is the reliance on self-report and performance-based measures that represent only a subset of the broader impulsivity construct (Warden et al. 2025). Future research incorporating a wider range of task types across impulsivity domains could provide a more comprehensive assessment and further expand upon the findings of this study. Finally, it should be noted that while participants were screened for major neurological and psychiatric conditions that would necessitate medications that could influence decision-making and impulsivity processes (e.g., SSRI/SNRI, Wellbutrin, Ritalin, Concerta), we did not systematically control all non-dopaminergic medications. Further research should consider more comprehensive documentation and control of medication types to verify our findings and rule out the potential effects of such medications.

To conclude, despite growing evidence, the cerebellum’s specific role in different impulsivity processes remains unclear. Influential neuroanatomic models of impulsivity have primarily focused on cortico-striatal circuits, often overlooking the cerebellum’s contributions (Miquel et al. 2019; Chen et al. 2022). The current study of the selective pattern of impulsivity in CA supports emerging theories of the cerebellum’s role in higher-order functions (Middleton and Strick 2000; Ravizza et al. 2006; Strick et al. 2009; Guell et al. 2018; Hull 2020; Saban et al. 2024). Our results align with previous research suggesting the cerebellum participates in reward processing and decision-making (Deverett and Oostland 2023; Zang and De Schutter 2023; Nicholas et al. 2023). These findings prompt further investigation of cerebellar contributions beyond motor control, supporting its inclusion in network-based models of impulsivity processes.

## Data Availability

All data needed to evaluate the conclusions in the paper are present in the paper. In support of open science initiatives, anonymized datasets, including group assignment, BIS-11 scores, and DD scores, could be available from the corresponding authors on reasonable request.

## Abbreviations

AUC: Area under the curve
BIS-11: Barratt Impulsiveness Scale
CA: Cerebellar ataxia
CAN: Center for Accessible Neuropsychology
DD: Delay discounting
FPR: False positive rate
L_1_: Lasso
LR: Logistic regression
MCQ-27: 27-Item Monetary Choice Questionnaire
ML: Machine learning
MoCA: Montreal Cognitive Assessment Test
NH: Neurotypical healthy
PD: Parkinson’s disease
RF: Random forest
ROC: Receiver operating characteristic
SARA: Scale for Assessment and Rating of Ataxia
TPR: True positive rate

## Footnotes

1 BIS-11 experiment: 21 SCA3, 1 ADCADN, 1 ARCA-1, 1 acquired, 1 Friederich’s, 1 CACNA1S, 1 CANVAS, 1 EA2, 1 Paraneoplastic, 6 SCA1, 1 SCA15, 5 SCA2, 2 SCA34, 1 SCA48, 3 SCA5, 13 SCA6, 4 SCA8, 1 Telangiectasia, 1 Mitochondrial, 2 SCA14, 3 SCA27B, 1 SCA36, 1 SCA45, 1 SCA50, 1 SCA7, and 65 with unknown subtype. DD experiment: 13 SCA3, 1 ADCADN, 1 ARCA-1, 1 acquired, 1 Friedreich’s ataxia, 1 CACNA1S, 1 EA2, 1 Paraneoplastic, 7 SCA1, 1 SCA15, 7 SCA2,1 SCA28, 2 SCA34, 1 SCA48, 3 SCA5, 17 SCA6, 4 SCA8, 1 SYNE1, 1 Telangiectasia, 1 Mitochondrial, 2 SCA14, 3 SCA27B, 1 SCA36, 1 SCA45, 1 SCA50, 1 SCA7, and 34 with unknown subtype.

## Code Availability

The code used for data analysis is available from the corresponding author upon reasonable request. The analysis scripts were developed using open-source R packages.
